# Prevalence of pelvic organ prolapse and related factors in a general female population

**DOI:** 10.4274/tjod.90582

**Published:** 2014-09-15

**Authors:** Hakan Aytan, Devrim Ertunç, Ekrem C. Tok, Osman Yaşa, Hakan Nazik

**Affiliations:** 1 Mersin University Faculty of Medicine, Department of Obstetrics and Gynecology, Mersin, Turkey; 2 Adana Numune Training and Research Hospital, Clinic of Obstetrics and Gynecology, Adana, Turkey

**Keywords:** Pelvic organ prolapse, POP-Q, prevalence, risk factors

## Abstract

**Objective:**

The aim of this study was to assess the prevalence and the related factors of pelvic organ prolapse (POP) in a female population to whom health care services are offered.

**Materials and Methods:**

1354 of the 3000 women admitted to the outpatient clinic between June 2008 and December 2008 were enrolled as they accepted to participate to the study. 34 of these patients with a history of previous hysterectomy and/or any kind of pelvic reconstructive surgery were excluded. Baseline characteristics, as well as medical and obstetric history of the patients were recorded. All women underwent vaginal examination to determine the degree of prolapse by pelvic organ prolapse quantification (POPQ) system. POP-Q stages ≥2 were defined as prolapse. Women with and without prolapse were compared. Regression analysis was used in order to determine the independent predictors.

**Results:**

Prolapse (stage ≥2) was detected in 358 patients (27.1%). Patients with prolapse were found to be significantly older and heavier. They had a higher waist to hip ratio and had a higher parity. Compared to women without prolapse, cesarean rate was significantly lower in women with prolapse (10.6% vs. 20.8%; p<0.001), and the mean birth weight of the babies of the women with prolapse was significantly higher (3584±574 vs. 3490±389 g, p=0.004). Prevalence of prolapse was found to be decreased as the level of education increased. Waist to hip ratio (OR:46.2, CI: 3.3-655, p=0.005), parity (OR:1.5, CI:1.3-1.7, p<0.001), vaginal delivery (OR:1.5, CI: 0.3-0.8, p=0.005), and menopausal status (OR:1.2, CI: 1.1-1.4, p=0.005) were found to be independent predictors of development of POP.

**Conclusion:**

In the present study, POP was found to be associated with waist to hip ratio, parity, vaginal delivery, and menopausal status.

## INTRODUCTION

Pelvic organ prolapse (POP) is defined as the descent of uterus and vaginal walls through vaginal canal. Pelvic organs move downwards due to anatomical and or functional deformities of the tissues that support pelvic organs. As a component of pelvic floor dysfunction, POP which is a common health problem affecting about 30% of the women between 20-59 years of age and more than half of the women over 50 years of age attending to the clinics is the most common surgical indication following hysterectomy^(1,2)^. The life-time risk of a woman for POP surgery is estimated to be 19% and the reoperation risk even with an appropriate surgery is about 30%^(3,4)^. It is not only an important health problem but also is an important extra burden to the health expenditures.

The natural progress of POP is not yet completely understood. Studies assessing the epidemiology of POP are limited because standardized measures that can objectively evaluate its presence or absence, degree or the impact of the associated symptoms had not been used^(5)^. The staging process of POP with physical examination and the diagnostic methods also differ. In many studies Baden-Walker Halfway and Women Initiative Staging systems had been used^(6,7)^. Even though pelvic organ prolapse quantification (POPQ) system that has been recently developed in order to standardize physical examination generated a common language among the clinicians, it has not gained wide spread all over the world^(8,9)^. Evaluation of the epidemiology and natural history of the disease is limited because POP has a subjective nature of associated symptoms, is a diagnosis determined by physical examination and following large populations with interval standardized pelvic examinations is expensive and has logistical difficulties^(10)^.

Some risk factors have been determined for POP. Advanced age, white race, menopause, some systemic diseases, obesity, vaginal delivery, smoking, chronic constipation and giving birth to large babies have been proposed as risk factors in various studies^(10)^. Most of these studies are from foreign countries and studies from Turkish population are scarce. The aim of this study was to assess the prevalence and the related factors of POP in a general women-population attending to our clinic in the city of Mersin to whom health care services are offered.

## MATERIALS AND METHODS

A total of 3.000 consecutive women who attended the university hospital between June 2008 and December 2008 were included in this prospective study. The study cohort was recruited from the hospital database. Brief information about the study, procedures and the nature of the questionnaires were explained to each patient. One thousand three hundred fifty-four women agreed to participate. Thirty-four women because they had undergone a kind of pelvic surgery (such as hysterectomy, anterior/posterior colporraphy, sacrospinous fixation, sacral colpopexy) were excluded. Medical histories were obtained with a standardized form designed to assess obstetric and gynecologic histories, chronic diseases (e.g., diabetes mellitus, hypertension, rheumatologic disorders, cardiac, pulmonary, gastrointestinal, and renal diseases) and prescriptions. Height and weight of the patients were measured on the day of interview. Body mass index (BMI) was calculated as weight in kilograms divided by height in meters squared. Waist-to-hip ratio (WHR) was calculated by dividing waist to hip. Each participant gave written informed consent, and the protocol was approved by the Local Ethical Committee on Research with Human Subjects.

Pelvic examination was performed to all of the patients by the investigators. The staging of pelvic organ prolapse was done with POP-Q (pelvic organ prolapse quantification) system conformed to the standards and terminology set forth by the International Continence Society^(8)^. POP-Q examination was performed while the patient was in dorsal lithotomy position. Subjects underwent the POP-Q examination in the dorsal lithotomy position. All points for the POP-Q examination, except for total vaginal length, were recorded at maximal protrusion with Valsalva maneuver. If the subject was not able to perform a Valsalva maneuver, she was first coached by the examiner in the performance of a Valsalva maneuver. If they still could not perform a Valsalva maneuver, the measurements were recorded with the subject forcefully coughing. An overall stage was assigned to each patient, according to the most severely prolapsing compartment. Women with stage 2 prolapse were considered as having genital prolapse^(11)^.

Statistical analysis was accomplished with SPSS (version 17, demo, SPSS Inc., Chicago, IL, USA). Data of women with POP and without POP were compared with student t test for normally distributed continuous variables and with chi square or Fisher’s exact tests for binary data. Logistic regression analysis was used to determine independent predictors of POP. A p value of <0.05 was considered as significant.

## RESULTS

The prevalence of genital prolapse was 27.1% in the assessed 1320 women. The general characteristics of the patients with and without prolapse are depicted in (Table 1). As shown in the table women with prolapse were significantly older, heavier, had an increased waist to hip ratio and had given more birth (Table 1). When we look at the mode of delivery, the rate of delivery with cesarean section was significantly lower in women with prolapse (10.6% vs 20.8%, p<0.001, respectively) and the mean birth weight of the women with prolapse was significantly higher when compared to the women without prolapse (3584±574 vs 3490±389 g, p=0.004) (Table 1). The level of education was found to be significantly lower in women with prolapse compared to women without prolapse (Table 1).

In order to find the independent predictors of POP a logistic regression analysis including age, BMI, WHR, parity, mode of delivery, menopausal status, presence of chronic diseases, smoking, level of education and yearly income was performed. According to this analysis especially waist-to-hip ratio, then parity, vaginal birth history and menopausal status were found to be the independent predictors that increase the risk of POP (Table 2).

## DISCUSSION

In order to devise optimal and cost-effective preventive and therapeutic strategies to deal with POP problem in a population, one should start by defining the prevalence of the condition and its risk factors in that population^(12)^. From that point a cross sectional population based study aimed to find the prevalence and the associated risk factors of POP was conducted in the population that health service was offered and it was found that increase in WHR and parity, giving vaginal birth and being in the menopause were independent predictors of POP development. Although not found to be independent predictors of POP development, patients with POP were significantly older, heavier, had higher maximum birth weight, had lower rate of cesarean section and had a lower education level when compared to women without POP.

There are limited studies that assessed the prevalence of POP in our country when the literature was reviewed. Çam et al used POP-Q system and reported stage ≥2 POP rate as 33% and 38% in Turkish women with and without episiotomy respectively in their study that they assessed the effect of mediolateral episiotomy on the pelvic floor^(13)^. In another study that assessed the validation of the prolapse-related quality of life questionnaire in a selected Turkish population, stage ≥2 POP was found in 123 of the 218 assessed women (56.2%)^(14)^. In the present study the prevalence of POP was found to be 27.1%. The differences in the results may be due to the methodological differences and the geographic differences of the assessed populations. Çam et al enrolled only parous women in their study whereas in the present study all the women including nulliparous were enrolled. And in Seven et al’s study there is a difference that may originate from the differences in the study’s inclusion criteria^(14)^. However, all these data support that POP is a common health problem in our country.

In literature some modifiable and non-modifiable risk factors have been defined for POP development^(10)^. Obesity, vaginal birth, parity, smoking, chronic straining and large infant size are among the modifiable risk factors; whereas, age, race, menopause / estrogen deficiency, chronic lung disease, connective tissue disorders and neuropathy constitute the non-modifiable risk factors^(10,15,16)^. All these risk factors cause POP by resulting in damage to the support of the pelvic floor^(17)^.

Vaginal delivery and parity were found to be independent modifiable risk factors for POP development which is parallel to the literature. In addition increase in waist-to-hip ratio was also found to be another modifiable risk factor for POP development in the present study. POP development following vaginal delivery is believed to result from structural disruption due to overstretching, compression and avulsions during childbirth and or secondary to denervation injury to the levator ani muscle^(5,18,19,20)^. Quiroz et al. reported that one vaginal delivery increases the risk of POP development 9.7 times (95% confidence interval: 2.68-35.35)^(21)^. Again a study from Italy reported that vaginal delivery compared with cesarean delivery increased POP risk 1.82 times (95% CI: 1.04-3.19)^(22)^. In the present study vaginal delivery was found to increase risk of POP development 1.5 times in the assessed population. The significant lower rate of POP in patients who delivered abdominally (10.6% vs 20.8%, p<0.001) is in line with these results. When we look at the parity as the second factor, in the epidemiological study of Oxford Family Planning Association parity was suggested to be the most important risk factor^(23)^. Similarly in the present study both vaginal and cesarean deliveries were considered as parity and parity was found to be a risk factor independent of the mode of delivery for development of POP. Levator ani muscle injury which is suggested to be a factor for development of POP and is more common during vaginal delivery, may be seen in both types of deliveries and therefore parity independent from delivery route comes out as an important risk factor for POP.

WHR is considered as an indicator of visceral obesity. Increased WHR was found to be an important risk factor for the development of POP in the assessed population. The relationship between WHR and POP has been also shown by Kudish et al. These investigators had shown that a change in WHR, evaluated in 0.1 increment decreases, was found to be associated with regression of both cystocele and rectocele^(24)^. The hypothesized mechanism is that this ratio is a reflection of larger mechanical forces directed toward the pelvic floor at rest or during cough or Valsalva maneuver and its reduction decreases the reflection of these forces to the pelvic floor^(24)^. BMI and the maximum birth weight did not come out to be independent predictors of POP development although they were found to be significantly higher in the women with POP compared to women without POP in the assessed population. Smoking was also not a risk factor.

Among the non-modifiable risk factors only menopausal status was found to be a risk factor for POP development and being in menopause was found to increase POP development risk 1.2 times. Atrophy that developed in the setting of estrogen deficiency after menopause is a concern for all the pelvic structures and as a result POP may develop. In addition, kyphotic changes due to osteoporosis that developed secondary to advanced age and estrogen deficiency causes a horizontal shift in the pelvic brim which results in reflection of the abdominal contents to the pelvic floor and urogenital hiatus rather than the pelvic brim^(25,26)^. Age has been reported to be an independent risk factor for the development of POP in many studies; however, in the present study although it was found that women with POP were significantly older, age itself did not come out to be a risk factor for POP development.

Giving birth with cesarean section was not found to be a preventive factor for the development of POP in the current study. However, this study has some limitations with regard to this issue. The women who gave birth with cesarean section after a vaginal delivery in their previous pregnancies were not assessed separately and it was impossible to assess because of significant recall bias how many of the women and how long did they experience labor before cesarean section. These factors must be kept in mind while interpreting the data about the preventive effects of cesarean section on the development of POP.

In conclusion in Turkey, the number of epidemiological studies on POP is very limited. In the present study it was aimed to assess the prevalence and the associated factors of POP in women admitting to our clinics and POP was found to be a common health problem in Turkish women that we offer service. Parity, WHR, vaginal delivery and menopausal status were found to be the independent predictors of POP development. To clarify the issue and to establish the possible geographical differences in our country, large epidemiological studies including different geographical places that will reflect the overall situation in Turkey are needed.

## Figures and Tables

**Table 1:**
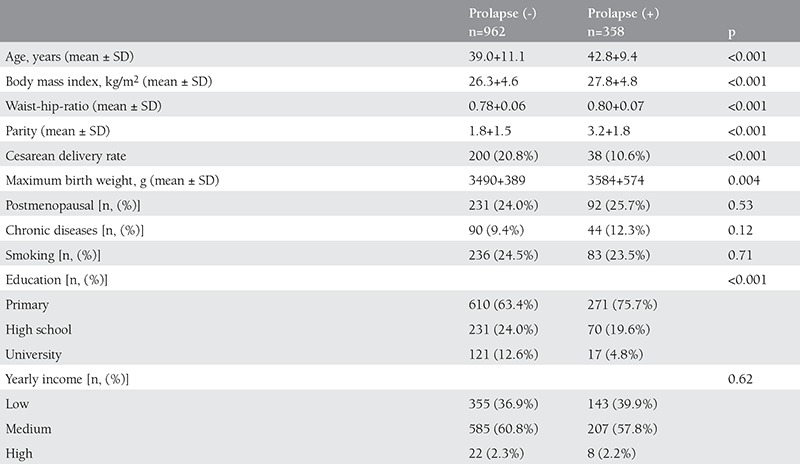
The general characteristics of women with and without genital prolapse

**Table 2:**
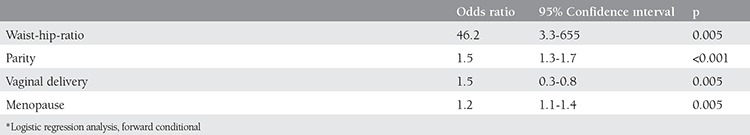
Multivariate analysis of the factors that affect pelvic organ prolapse*
